# *In vitro* Characterization of Gut Microbiota-Derived Bacterial Strains With Neuroprotective Properties

**DOI:** 10.3389/fncel.2019.00402

**Published:** 2019-09-20

**Authors:** Suaad Ahmed, Alessandro Busetti, Parthena Fotiadou, Nisha Vincy Jose, Sarah Reid, Marieta Georgieva, Samantha Brown, Hayley Dunbar, Gloria Beurket-Ascencio, Margaret I. Delday, Anna Ettorre, Imke E. Mulder

**Affiliations:** ^1^4D Pharma Research Ltd., Aberdeen, United Kingdom; ^2^School of Medicine and Dentistry, Institute of Medical Sciences, Foresterhill, University of Aberdeen, Aberdeen, United Kingdom

**Keywords:** gut microbiota-derived bacterial strains, microbiome, neurodegenerative diseases, neuroinflammation, neuroprotection, oxidative stress, gut-brain axis, short-chain fatty acids

## Abstract

Neurodegenerative diseases are disabling, incurable, and progressive conditions characterized by neuronal loss and decreased cognitive function. Changes in gut microbiome composition have been linked to a number of neurodegenerative diseases, indicating a role for the gut-brain axis. Here, we show how specific gut-derived bacterial strains can modulate neuroinflammatory and neurodegenerative processes *in vitro* through the production of specific metabolites and discuss the potential therapeutic implications for neurodegenerative disorders. A panel of fifty gut bacterial strains was screened for their ability to reduce pro-inflammatory IL-6 secretion in U373 glioblastoma astrocytoma cells. *Parabacteroides distasonis* MRx0005 and *Megasphaera massiliensis* MRx0029 had the strongest capacity to reduce IL-6 secretion *in vitro*. Oxidative stress plays a crucial role in neuroinflammation and neurodegeneration, and both bacterial strains displayed intrinsic antioxidant capacity. While MRx0005 showed a general antioxidant activity on different brain cell lines, MRx0029 only protected differentiated SH-SY5Y neuroblastoma cells from chemically induced oxidative stress. MRx0029 also induced a mature phenotype in undifferentiated neuroblastoma cells through upregulation of microtubule-associated protein 2. Interestingly, short-chain fatty acid analysis revealed that MRx0005 mainly produced C1-C3 fatty acids, while MRx0029 produced C4-C6 fatty acids, specifically butyric, valeric and hexanoic acid. None of the short-chain fatty acids tested protected neuroblastoma cells from chemically induced oxidative stress. However, butyrate was able to reduce neuroinflammation *in vitro*, and the combination of butyrate and valerate induced neuronal maturation, albeit not to the same degree as the complex cell-free supernatant of MRx0029. This observation was confirmed by solvent extraction of cell-free supernatants, where only MRx0029 methanolic fractions containing butyrate and valerate showed an anti-inflammatory activity in U373 cells and retained the ability to differentiate neuroblastoma cells. In summary, our results suggest that the pleiotropic nature of live biotherapeutics, as opposed to isolated metabolites, could be a promising novel drug class in drug discovery for neurodegenerative disorders.

## Introduction

Neurodegenerative diseases (NDDs) such as Parkinson’s disease (PD) and Alzheimer’s disease (AD) are disabling, incurable disorders, often associated with aging. Progressive and irreversible loss of neurons, neuroinflammation and degeneration of cellular functions and structures often associated with oxidative stress, are common features of NDDs (Habib et al., 2018). NDDs have a profound impact on the life expectancy of individuals and the socio-economy of the world population (Group, 2017). In recent years, there has been mounting interest in the gut microbiota and its interaction with the immune and central nervous system (CNS) (Fung et al., 2017). Alterations of the microbiota composition in neurodegenerative diseases including PD and AD have been well documented (Gerhardt and Mohajeri, 2018; Sun and Shen, 2018). Shifts in gut bacterial populations, possibly due to environmental and internal stress factors, can contribute to inflammation by altering the production of key signaling molecules in the host. Conversely, changes in host physiology can also affect the composition and diversity of the gut microbiota (Hollister et al., 2014; Fung et al., 2017).

The gut microbiota exerts important effects on brain functions and can affect physiological processes such as host stress responses (Cryan and Dinan, 2012; Foster and McVey Neufeld, 2013), behavior related to depression and anxiety (Foster and McVey Neufeld, 2013) and even susceptibility to autism (Hsiao et al., 2013; Foster et al., 2017). Gut bacteria can also exert neuroprotective effects, for instance by producing metabolites used by the host as neurotransmitters. About 95% of the serotonin in the human body is produced in the digestive tract and plays an important part in gut motor function and digestion, as well as in various cognitive and mood disorders (Yano et al., 2015). In mice, it has been shown that a particular subset of gut bacteria directly stimulates synthesis and release of intestinal serotonin (Yano et al., 2015). Germ-free mice produce lower than normal levels of dopamine and adrenaline, two important molecules involved in the response of the CNS to stress (Asano et al., 2012). Many gut bacteria can produce short-chain fatty acids (SCFAs) from dietary fiber. A reduction in SCFA concentration in fecal samples of PD patients has been described recently, highlighting a potential link between these metabolites and neurological disease (Unger et al., 2016).

The vagus nerve serves as a direct communication path between gut bacteria and the central nervous system (CNS). Recent publications indicate that the effects of a *Lactobacillus rhamnosus* strain on the CNS seem to depend on signals relayed by the vagus nerve (Bravo et al., 2011) and that gut commensal bacteria can influence anxiety-like symptoms in a mouse model of colitis only if the vagus nerve remains functionally intact (Bercik et al., 2011). The CNS and gastrointestinal (GI) tract also communicate through humoral and cellular mediators via the hypothalamic-pituitary-adrenal axis and the immune system by means of cytokines, chemokines and small peptides (De Palma et al., 2014).

Notwithstanding the different cell types and etiology associated with NDDs, inflammation is a major contributing factor to disease (Stephenson et al., 2018). Peripheral immune cells can contribute to neuroinflammation through the production of pro-inflammatory cytokines that are able to cross the blood-brain barrier (BBB) and activate microglia cells. Moreover, when the BBB is disrupted, the brain parenchyma can be exposed to pathogens and immune cells (Hirsch and Hunot, 2009; Zhan et al., 2018).

Stress stimuli can increase gut permeability, which in turn facilitates translocation of gut bacteria and immune responses in the gut mucosa (Keita and Soderholm, 2010). Gram-negative bacteria in the gut can release cell membrane components such as lipopolysaccharide (LPS), which can engage Toll-like receptor 4 (TLR4) on host cells and trigger a pro-inflammatory response (Rakoff-Nahoum et al., 2004). Low levels of circulating LPS can compromise both passive and active BBB mechanisms, rendering the CNS vulnerable to neurotoxic substances and activated immune cells from the periphery (Varatharaj and Galea, 2017). TLR activation by pathogen-associated molecular patterns (such as LPS) and damage-associated molecular patterns (e.g., α-synuclein in PD) is a dynamic process. TLR activation triggers a series of downstream molecular pathways leading to the translocation of NF-κB to the nucleus and culminating in upregulation of pro-inflammatory cytokine expression. Therefore, therapeutic interventions aimed at interfering with TLR signaling could decrease pro-inflammatory cytokine responses leading to an overall reduction of neuroinflammation, oxidative stress and neuronal death (Fellner et al., 2013; Rietdijk et al., 2016).

We have identified gut microbiota strains that possess modulatory activity on human cell biology and physiology readouts relevant to neurodegeneration and neuroinflammation which may then be developed as Live Biotherapeutics. Here, we describe the *in vitro* characterization of two gut bacterial strains with potential neuroprotective properties, namely *Parabacteroides distasonis* MRx0005 and *Megasphaera massiliensis* MRx0029, and report their ability to modulate both neuroinflammation and barrier function *in vitro*.

## Materials and Methods

### Cells and Reagents

Trypsin/EDTA, sterile phosphate buffered saline (PBS), Dulbecco’s modified Eagle’s medium (DMEM with 4.5 g/L D-glucose), Minimum Essential Medium Eagle (MEME), Nutrient Mixture F-12 Ham, heat inactivated Foetal Bovine Serum (FBS), L-Glutamine, penicillin, streptomycin, retinoic acid, 4′,6-diamidino-2-phenylindole (DAPI), HPLC-grade diethyl ether, formic acid, acetic acid, propionic acid, butyric acid, hexanoic acid and phosphoric acid were purchased from Sigma-Aldrich (Millipore, United Kingdom).

hIL-6 and hIL-8 ELISA kits were purchased from Peprotech (London, United Kingdom).

HPLC-grade hexane, ethyl acetate, acetonitrile, water and methanol, Dichloro-fluorescein diacetate (DCFDA), BCA protein assay kit and general tissue culture plasticware (Corning) were purchased from Fisher Scientific (Loughborough, United Kingdom).

HEK293 reporter cells stably expressing human TLR4 were purchased from InvivoGen, U373 cells from Sigma-Aldrich, SH-SY5Y cells and HT29-MTX-E12 (HT29-mtx) from the European Collection of Authenticated Cell Cultures (ECACC, Public Health England, Salisbury, United Kingdom).

Valeric acid, monoclonal anti-MAP2 (sc-74421) and anti-β3-tubulin (clone 2G10, sc-80005) antibodies were purchased from Santa Cruz Biotechnology.

Anti-β-actin antibody (ab1801) and Alexa Flour 594 conjugated Phalloidin (ab176757) were purchased from Abcam (United Kingdom).

### Bacterial Culture – Collection of Bacterial Cell-Free Supernatants

A total of 50 proprietary bacterial strains from the 4D Pharma Ltd., culture collection, a strain library consisting of isolates derived from fecal samples of healthy human donors, were grown anaerobically in Yeast Casitone Fatty Acids^+^ broth media (YCFA^+^, E&O Labs, Scotland, United Kingdom) (Yuille et al., 2018). Viable cell counts (VCC) were determined using an Automated Spiral Plater (Don Whitley Scientific, Bingley, United Kingdom) and a Protocol 3 Completed Automated Colony Counting and Chromogenic Identification System (Synbiosis, Cambridge, United Kingdom). Bacterial cell-free supernatants (BCFS) were obtained from stationary phase cultures (inoculated from an overnight culture of a subbed colony from a streaked freezer stock) by centrifuging 10 ml of cultures at 5000 × *g* for 5 min and filtering using a 0.2 μM filter (Millipore, United Kingdom). 1 ml aliquots of the bacterial cell-free supernatants were stored at −80°C until use.

### Preparation of MRx0005 and MRx0029 Cultures

Strains MRx0005 and MRx0029 were cultured to stationary phase as described above in a total of 100 ml of YCFA^+^ media. BCFS were prepared as described above. 20 ml aliquots of each BCFS (untreated control) were stored at −80°C until needed for sequential extraction.

### Sequential Solvent Extractions – Preparation of Crude Extracts of MRx0005 and MRx0029

Three biological replicates of MRx0005 and MRx0029 BCFSs and YCFA^+^ (media control) were extracted sequentially with HPLC-grade hexane (HEX), diethyl ether (DE), ethyl acetate (EtOAc), acetonitrile (ACN) and methanol (MeOH). Briefly, 20 ml of BCFS were placed in glass vials and extracted at room temperature (RT) in 20 ml of HEX on a rotary shaker (70 rpm) for 30 min. A total of three extractions were performed on each BCFS and YCFA^+^ media control. The remaining aqueous layers were then extracted at RT in 20 ml of DE, EtOAc on a MX-RD-Pro rotary shaker (70 rpm) for 30 min a total of three times. The combined extracts of each sample were dried under reduced pressure in an R-300 rotary evaporator equipped with a V-300 vacuum pump (Büchi, Flawil, Switzerland) at a temperature not exceeding 30°C. The resulting extracts were re-solubilized in 2 ml of corresponding solvent and aliquoted in four 1.5 ml Eppendorf tubes (500 μl each corresponding to 5 ml of original sample). The remaining aqueous layers were then extracted at RT in 20 ml of DE, EtOAc on a MX-RD-Pro rotary shaker (70 rpm) for 30 min a total of three times. The combined extracts of each sample were dried under reduced pressure in a R-300 rotary evaporator equipped with a V-300 vacuum pump (Büchi, Flawil, Switzerland) at a temperature not exceeding 30°C. The resulting extracts were re-solubilized in 2 ml of corresponding solvent and aliquoted in four 1.5 ml Eppendorf tubes (500 μl each corresponding to 5 ml of original sample).

The remaining aqueous layers were evaporated to dryness using an R-300 rotary evaporator. The resulting dry extracts were extracted for 30 min in 20 ml of ACN a total of three times. The ACN extracts were combined, evaporated to dryness using a rotary evaporator, resolubilised in 2 ml of ACN and aliquoted in four 1.5 ml Eppendorf tubes (500 μl each). The remaining dry extracts (ACN insoluble portion of the extracts) were then extracted for 30 min in 20 ml of MeOH a total of three times. The MeOH extracts were combined, evaporated to dryness using an R-300 Rotary Evaporator, resolubilised in 2 ml of MeOH and aliquoted in four 1.5 ml Eppendorf tubes (500 μl each).

Aliquots of the crude extracts were kept overnight at −20°C inducing the precipitation of proteinaceous components. Following overnight precipitation, each aliquot was centrifuged at 10,000 × *g* for 6 min and transferred to a new 2 ml tube. Overnight precipitation was repeated three times after which extracts were dried in a RVC 2-18 CDPlus speedvac (Christ, Osterode am Harz, Germany) and weighed. All dried aliquots of each extract were stored at −80°C until further use.

### SCFA Extraction From Bacterial Cell-Free Supernatants

Short-chain fatty acid extraction from YCFA^+^, YCFA^+^ spiked with a standard mix of SCFAs (40 mM acetic acid and 20 mM formic acid, propionic acid, butyric acid, valeric acid and hexanoic acid) as well as the methanol extracts of MRx0005 and MRx0029 and YCFA^+^ (media control) was conducted according to the method of De Baere et al. (2013).

### HPLC Analysis of SCFAs

HPLC detection and quantification of SCFAs was conducted according to the method of De Baere et al. (2013) with slight modifications. Briefly, HPLC analysis was performed using a Waters e2695 HPLC system equipped with a Waters Photodiode Array (PDA) detector 2998 (Waters Limited, Elstree, United Kingdom). HPLC analysis of SCFAs standards, SCFAs extracted from MRx0005 and MRx0029 BCFS and MRx0005 and MRx0029 hexane, diethyl ether, ethyl acetate, acetonitrile and methanol extracts were performed using an Xselect^®^ HSS T3 3.5 μm × 4.6 mm × 150 mm LC column (Waters Limited, Elstree, United Kingdom). The LC analysis was performed using the photodiode array detector (PDA) set to analyse wavelengths of 200–800 nm. SCFA detection and quantification was performed at 210 nm. The mobile phase consisted in 25 mM sodium phosphate buffer in HPLC water (pH adjusted to 3.0 using phosphoric acid (A) and acetonitrile (B). The LC method for SCFA detection and quantification was run using the solvent system with the following gradient: t0′ A = 95%, B = 5%; t10′ A = 95%, B = 5%; t30′ A = 30%, B = 70%; t31′ A = 0%, B = 100%; t36′ A = 0%, B = 100%; t38′ A = 5%, B = 95%; t60′ A = 5%, B = 95%; flow = 1 ml/min.

A seven-point calibration curve was prepared for each SCFA by injecting 20 μl of a two-fold serial dilution of a SCFA (40 mM acetic acid and 20 mM formic acid, propionic acid, butyric acid, valeric acid and hexanoic acid). Qutification- extraction efficiency was calculated using the formula below:

$$\left[\mathrm{SCFA}\mathrm{in}{\mathrm{YCFA}}^{+}\mathrm{spiked}\mathrm{and}\mathrm{extracted}\right]/[\mathrm{SCFA}\mathrm{in}{\mathrm{YCFA}}^{+}\mathrm{spiked}\mathrm{not}\mathrm{extracted}]$$

Extraction efficiency was used to determine the concentrations of individual SCFAs in each sample. The production of specific SCFAs was calculated by subtracting the amount of corresponding SCFA present in the unspiked media control.

### Targeted Metabolomics: Bacterial Metabolites and Fatty Acid Analysis

Sample analysis was carried out by MS-Omics (Copenhagen, Denmark). A mixed pooled sample (QC sample) was created by taking an aliquot from each sample. This sample was analyzed with regular intervals throughout the sequence. Matrix effects were tested for quantified compounds by spiking the QC sample in a minimum of two levels.

For GC-metabolite analysis, samples were derivatized with methyl chloroformate using a slightly modified version of the protocol described by Smart et al. (2010). All samples were analyzed in a randomized order. Analysis was performed using GC (7890B, Agilent) coupled with a quadrupole detector (59977B, Agilent). Raw data was converted to netCDF format using Chemstation (Agilent), before the data was imported and processed in Matlab R2014b (Mathworks, Inc.) using the PARADISe software described by Johnsen et al. (2017).

For SCFA analysis, samples were acidified using hydrochloric acid, and deuterium-labeled internal standards were added. Analysis was performed using a high-polarity column (Zebron^TM^ ZB-FFAP, GC Cap. Column 30 m × 0.25 mm × 0.25 μm) installed in a GC (7890B, Agilent) coupled with a quadrupole detector (59977B, Agilent). Raw data was converted to netCDF format using Chemstation (Agilent), before the data was imported and processed in Matlab R2014b (Mathworks, Inc.) using the PARADISe software described by Johnsen et al. (2017).

### Quantification of Bacterial Indole Production From L-Tryptophan

Bacterial Indole production was quantified using an assay described previously (Sasaki-Imamura et al., 2010). Bacteria were cultured to stationary phase of growth. 0.5 mM indole in YCFA^+^ media was used as a positive chemical control in this assay. The Indole assay was performed using 24-well (non-treated) assay plates. 100 mM tryptophan solution in HCl was dispensed into each well to give a final concentration of 6 mM. 1 ml stationary phase bacterial culture was added to each well and incubated for a further 48 h. Assay plates were centrifuged at 3,500 × *g* at RT for 10 min. The supernatant was retained, and the pellet discarded. In a 96-well plate 140 μl supernatant was dispensed in triplicate. 140 μl Kovac’s reagent was added and the absorbance read at 540 nm using a BioRad iMark microplate absorbance reader. The standard curve was prepared by plotting absorbance as a function of final Indole concentration (mM). Indole concentration of the test sample was calculated using the equation extrapolated from linear regression of the standard curve.

### 2,2-Diphenyl-1-Picrylhydrazyl (DPPH) Free-Radical Assay

BCFS were thawed at 4°C for approximately 2 h prior to use. All samples were diluted 1:2 in 1.5 ml microfuge tubes using sterile 5 mM PBS pH 7 yielding a final volume of 1 ml. A stock solution of 500 μM Trolox in 5 mM PBS, pH 7 was prepared to make the standard curve. Lazaroid antioxidant U83836E was included (200 μM in 100% methanol) as a positive control. The DPPH assay was performed in a 96-well plate as described previously (Chai et al., 2012) with minor modifications made. In brief, 10 μl sample/standard/control was added, in triplicate, to corresponding wells of a 96-well plate. 200 μl of a 200 μmol/L DPPH solution was added to three empty wells as a control. 190 μl of 200 μmol/L DPPH was added to sample/standard/control wells and plates incubated in the dark for 30 min at RT. Absorbance was read at 515 nm using a BioRad iMark microplate absorbance reader. DPPH radical scavenging activity was calculated as follows:

DPPHradicalscavengingactivity(%)=[1-(A-sampleA)blank/A)control]×Dilutionfactor×100

where A_sample_ was the average absorbance of sample + 200 μmol/L DPPH, A_control_ was the average absorbance of methanol DPPH without sample, and A_blank_ is the average absorbance of YCFA^±^ media blank.

### 2,2′-Azino-Bis-3-Ethylbenzthiazoline-6- Sulfonic Acid (ABTS) Assay

The total antioxidant capacity assay was performed using the Antioxidant Assay Kit according to the manufacturer’s instructions. Briefly, all samples were diluted 1:4 in 1X assay buffer. In a 96-well plate, 10 μl standard/control/sample was added in triplicate. 20 μl myoglobin working solution was added to all standard/control/sample wells. 150 μl ABTS substrate solution was added to each well and the absorbance measured at 405 nm using a BioRad iMark microplate absorbance reader.

### Bacterial Cell-Free Supernatants Used in Cell-Based Assays

10% v/v of BCFS was used throughout all the cell-based assays presented in this study. During the assay development stage, a range of YCFA^+^ volumes across all assay platform was tested for interference. At 10% v/v, YCFA^+^ media did not affect *per se* the different biochemical and cellular read-outs. YCFA^+^, the bacterial growth media considered our blank, is a rich media containing amino-acids, vitamins and SCFAs, so it is expected to interfere at some level with biochemical and cellular assays. Moreover, the results from metabolomics analysis further support the use of 10% v/v as the right compromise to allow a clear appreciation of the effects attributable to the different bacterial metabolites. A concentration of BCFS higher than 10% v/v would have altered the pH of the culture media with dramatic effects on cell viability or would have overly activated the cells in our *in vitro* assays.

### Reporter Assay for HEK-Blue hTLR4

HEK293-Blue reporter cells stably expressing human TLR4 (HEK-TLR4), were cultured according to the manufacturer’s instructions. Briefly, HEK-TLR4 cells were maintained in DMEM 4.5 g/L D-glucose supplemented with 10% (v/v) heat-inactivated FBS, 4 mM L-Glutamine, 100 U/ml penicillin, 100 μg/ml streptomycin, 100 μg/ml normocin, 1X HEK-Blue selection media.

Briefly, cells were washed with PBS, dissociated in PBS and collected in growth media. Cells were plated in 96-well plates at a density of 25,000 cells/well. To evaluate the effect of bacteria strains on LPS inducing NF-κB promoter activation, cells were treated with 10 ng/ml LPS in presence or absence of 10% supernatants and incubated in a CO_2_ incubator. Treatments proceeded for 22 h at 37°C and 5% CO_2_, after which the detection of Secreted Embryonic Alkaline Phosphatase (SEAP) activity from cell culture supernatant was performed using QUANTI-blue solution according to manufacturer’s instructions. Briefly, 20 μl of cell-free supernatant was collected and analyzed for the presence of SEAP by mixing with 200 μl of sterile-filtered QUANTI-Blue detection media. After 2 h incubation at 37°C, optical density was measured at 655 nm on a microplate reader (iMark microplate, Bio-Rad).

### U373 Cell Treatment

U373 is a human glioblastoma astrocytoma cell line. Cells (passage 20th–37th) were maintained in 25 ml MEME supplemented with 10% heat-inactivated FBS, 4 mM L-Glutamine, 100 U/ml penicillin, 100 μg/ml streptomycin and 5 μg/ml plasmocin, 1% Non-Essential Amino Acids, 1% Sodium Pyruvate (referred to throughout as full growth media).

Cells were plated in 24-well plates at a density of 100,000 cells/well in 1 ml of full growth media and left to rest at 37°C and 5% CO_2_ for 72 h.

#### BCFS Treatment

On the day of the treatment, the media was removed from each well, cells were rinsed with 0.5 ml wash media (serum free MEME), 0.9 ml stimulation media (MEME media containing 2% FBS) containing 1 μg/ml LPS was added to the appropriate wells and incubated at 37°C and 5% CO_2_. After 1 h pre-incubation, cells were removed from CO_2_ incubator and treated with 100 μl bacteria supernatant. Media control was used as control.

#### SCFA Treatment

Cells were pre-treated for 1 h with 1 μg/ml LPS indicated above and incubated at 37°C and 5% CO_2_. After 1 h pre-incubation, cells were removed from CO_2_ incubator and treated with increasing concentration of fresh prepared Sodium Butyrate (SB), Sodium Valerate (SV) and Hexanoic Acid (HA).

#### MRx0005 and MRx0029 Fraction Treatment

Cells were pre-treated for 1 h with 1 μg/ml LPS as indicated above. Afterward, cells were removed from CO_2_ incubator and treated with 100 μl of the different fractions. Fractions from media were used as controls.

Following each treatment, cells were incubated for 24 h incubation at 37°C and 5% CO_2_. Afterward cell-free supernatants were collected and centrifugated at 10,000 × *g* at 4°C for 3 min. Samples were aliquoted in 1.5 ml microtubes and stored in −80°C for hIL-6 and hIL-8 ELISA.

### HMC3 Cell Treatment

Human microglia HMC3 cells were grown in glutamine-supplemented EMEM media containing 15% heat inactivated FBS and 100 U/ml penicillin and 100 μg/ml streptomycin. HMC3 cells were plated in 24 well plates at a density of 50,000 cells/well. Cells were left in CO_2_ incubator to rest for 48 h. The cells were then washed in blank EMEM and pre-treated in 2% FBS growth media with 10 ng/ml TNF-α for 1 h. Thereafter 10% cell-free bacterial supernatants for MRx0005 and MRx0029 stationary growth cultures were added to TNF-α-treated and untreated wells and incubated in CO_2_ incubator at 37°C for 24 h. Cell-free supernatants were collected and centrifugated at 10,000 × *g* for 3 min and 4°C. Samples were aliquoted in 1.5 ml microtubes and stored in −80°C for hIL-6 and hIL-8 ELISA.

### Measurement of IL-6 and IL-8 From U373 and HMC3 Cells

Secretion of IL-6 and IL-8 was analyzed using hIL-6 and hIL-8 Standard ELISA Kits, according to the manufacturer’s protocol in the cell-free supernatants from U373 and HMC3 cells treated as described above. Samples were measured at 405 nm with correction wavelength set at 655 nm on a microplate reader (iMark, Bio-Rad). Raw data were plotted and analyzed using GraphPad Prism 7 software. The hIL-6 ELISA development kit allows the quantitative measurement of human IL-6 within the range of 24-1500 pg/ml, while hIL-8 ELISA kit the range is 15.6-1000 pg/ml.

### ROS Measurement in U373, HMC3, and SH-SY5Y Cells

To evaluate ROS production, U373 cells and HMC3 were plated in black 96 well plates at a density of 10,000 cells/well. U373 cells were rested for 72 h while HMC3 were left to rest for 48 h. Cells were washed with pre-warmed PBS and stained with 10 μM DCFDA molecular probe for 20 min in growth medium containing 2% FBS. Afterward, the cells were washed with pre-warmed PBS again and treated with 100 μM TBHP in the presence or absence of 10% BCFS for 2 h.

Neuroblastoma SH-SY5Y cells were grown in 50% MEM and 50% Nutrient Mixture F-12 Ham media supplemented with 2 mM L-Glutamine, 10% heat-inactivated FBS, 100 U/ml penicillin and 100 μg/ml streptomycin. Cells were plated in growth medium on a black 96-well plates at 5,000 cells/well and placed in CO_2_ incubator. After 24 h, media was replaced with differentiation medium (growth medium containing 1% FBS) and 10 μM retinoic acid (RA). Differentiation medium was replaced every other day and cells were used after 10 days of differentiation. On Day 10, cells were washed with pre-warmed PBS and stained with 10 μM DCFDA molecular probe for 20 min in growth medium containing 1% FBS. Then, cells were washed with pre-warmed PBS again and treated with 100 μM TBHP in the presence or absence of 10% BCFS for 2 h. After the different treatments, plates were examined under light microscope for obvious changes in cell morphology (e.g., round cells, irregular shape, cell detachment). No dramatic changes in the morphology were observed in any of the cell lines used.

Fluorescence intensity was measured using a TECAN plate reader at Excitation 485 nm/Emission 530 nm. Raw data were plotted and analyzed using GraphPad Prism 7 software.

### Gene Expression in SH-SY5Y Cells

SH-SY5Y were plated in 6-well plates at a density of 0.5 × 10^6^ cells. After 24 h, cells were treated in differentiation medium (growth medium containing 1% FBS without RA) with 10% BCFS or YCFA^+^ or 10 μM RA for 24 h. Thereafter, representative images were taken using a phase contrast EVOS XL core microscope at 40×/0.65 magnification. Cells were collected, and total RNA was isolated according to the RNeasy mini kit protocol (Qiagen). cDNA was made using the High Capacity cDNA reverse transcription kit (Applied Biosystems). Gene expression was measured by qPCR. GAPDH was used as internal control. Fold change was calculated according to the 2^–ΔΔCt^ method. Primer sets are listed in Supplementary Table S1.

### Immunolabelling and Cell Imaging in SH-SY5Y Cells

Cells were seeded onto 8-well chamber slides (Marienfeld Laboratory Glassware) at 5 × 10^4^ cells/well overnight and treated with 10% BCFS for 24 h. Afterward, cells were fixed with 4% paraformaldehyde in PBS (pH 7.3) for 20 min at RT. Fixed cells were washed with PBS, and permeabilized with 1% Triton X-100 in PBS for 10 min. After washing with PBS, the slides were incubated with blocking buffer (4% BSA/PBS) for 1 h at RT before adding anti-MAP2 antibody or β3-tubulin antibodies diluted in 1% BSA/PBS for 1 h at 4°C. They were then washed twice with PBS, followed by incubation with Alexa Fluor-488 conjugated anti-mouse antibody and Alexa Fluor-594 conjugated Phalloidin for 1 h at RT. After washing 3X with PBS, slides were labeled with DAPI and mounted with Vectashield^®^ (Vector Laboratories). Slides were viewed using an Axioskop 50 microscope (Zeiss) equipped with a 63×/1.2 W Korr objective and filter sets suitable for detection of the fluorochromes used. Manual exposure times for the digital acquisition of images immuno-labeled with MAP-2 were kept constant allowing comparison between different wells and treatments. Phalloidin (F-actin) and DAPI (nuclei) exposure times varied to suit the field of view. Randomized fields of view were acquired using a QImaging camera controlled by Image Pro Plus software. Images were saved as TIFF files and opened in Adobe Photoshop CC 2015.1.2. Images of MAP2, DAPI and Phalloidin were then overlaid and merged. Representative images were selected to illustrate the differences in abundance and location of the proteins examined.

### Immunoblotting in SH-SY5Y Cells

SH-SY5Y cells were cultured under the indicated conditions described above, treated with MRx0005 and MRx0029 for 24 h and lysed in RIPA buffer containing a cocktail of protease inhibitors (Roche Diagnostics, United Kingdom). Protein concentration was estimated using the BCA protein assay kit, separated by SDS-PAGE and transferred to a PVDF membrane. Membranes were then blocked with 5% non-fat dry milk or 5% BSA and incubated overnight at 4°C with the primary antibodies (MAP2 and β3-tubulin, respectively). The blots were then incubated with the appropriate horseradish peroxidase (HRP)-conjugated secondary antibody, and proteins were detected by chemiluminescence detection kit (Pierce Biotechnology, Rockford, IL, United States). For both MAP2 and β3-tubulin, β-actin served as a control to monitor protein loading variability amongst samples.

### Statistical Analysis

Normally distributed data are presented as mean ± SEM; One-way Anova (Sidak’s multiple comparison test) was used to analyse the data presented in this paper. A *p* value < 0.05 was deemed significant in all cases.

## Results

### *In vitro* Cytokine Modulation After Exposure of a Glioblastoma Astrocytoma Cell Line to Different Bacterial Strains

**FIGURE 1:**
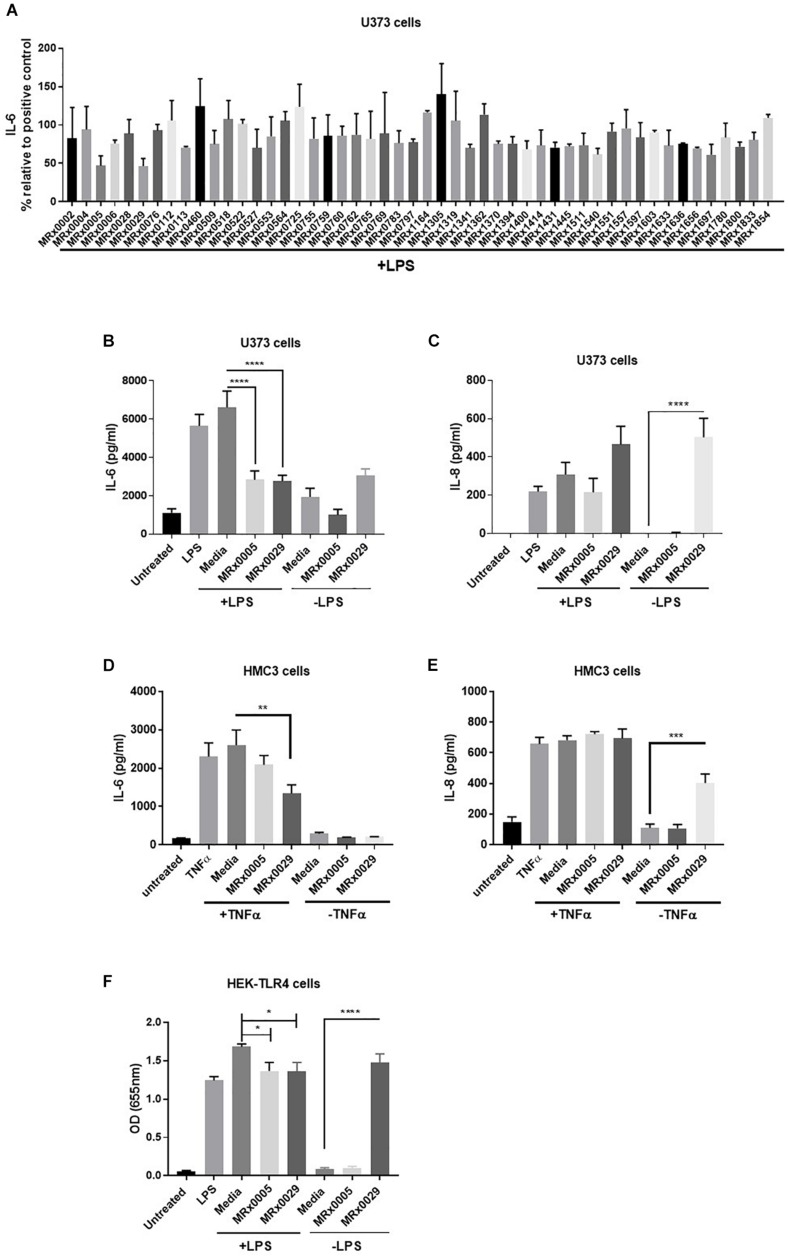
Differential mode of action between MRx0005 and MRx0029: implication for neuroinflammation. U373 cells were pre-treated with 1 μg/ml LPS followed by 10% bacterial cell-free supernatants (BCFS) or media alone. Cell-free supernatants were collected 24 h after treatment from U373 cells and secretion of IL-6 was measured by ELISA. Data are mean ± SEM (*n* = 2) **(A)**. U373 cells were treated with 1 μg/ml LPS followed by 10% BCFS from MRx0005 and MRx0029 and secretion of IL-6 **(B)** and IL-8 **(C)** measured by ELISA. Controls included also cells treated with BCFS alone. Data are mean ± SEM (*n* = 5 for IL-6, *n* = 4 for IL-8). HMC3 cells were pre-treated with TNF-α and then with 10% BCFS from MRx0005 and MRx0029 or media alone for 24 h and IL-6 **(D)** and IL-8 **(E)** measured by ELISA. Controls included also cells treated with BCFS alone. Data are mean ± SEM (*n* = 3). **(F)** HEK-Blue hTLR4 cells were treated with 10 ng/ml LPS in the presence of 10% BCFS from MRx0005 and MRx0029 or media as control. Controls included also cells treated with BCFS alone. After 22 h, NF-κB-induced SEAP activity was measured using QUANTI-Blue at 655 nm. Data are mean ± SEM (*n* = 5).

As neuroinflammation plays a pivotal role in neurodegenerative diseases (NDDs), the stationary phase bacterial cell-free supernatants (BCFS) of 50 bacterial strains from our proprietary MicroRx (MRx) culture collection were screened for their capacity to induce an anti-inflammatory response in the U373 glioblastoma astrocytoma cell line after treatment with lipopolysaccharide (LPS). After 24 h of treatment, secretion of the pro-inflammatory cytokine IL-6 was measured by ELISA. U373 cells secreted a significant amount of IL-6 in response to LPS in the presence of 2% serum, which provides the soluble form of CD14, the co-receptor for TLR4 (Flo et al., 2002) (Figure 1). Out of the fifty strains tested, MRx0005 (a *P. distasonis* strain) and MRx0029 (a *M. massiliensis* strain) displayed the strongest reduction of IL-6 in U373 cells after treatment with LPS (Figure 1A) and were therefore selected for further investigation of their potential neuroprotective profile.

### *In vitro* Cytokine Modulation of Multiple Brain-Derived Cell Lines After Exposure to MRx0005 and MRx0029

U373 cells were again treated with LPS, and secretion of IL-6 and IL-8 was measured. BCFS from stationary phase cultures of both MRx0005 and MRx0029 significantly down-regulated the production of IL-6 in U373 cells pre-treated with LPS (Figure 1B). Although not significant, MRx0029 alone slightly increased the basal level of IL-6 compared to the media alone. Interestingly, a different signature for the two strains was observed for IL-8 secretion by U373 cells. MRx0005 decreased the amount of LPS-induced IL-8, while only negligible levels of IL-8 were produced in the absence of LPS (Figure 1C). On the contrary, MRx0029 induced higher IL-8 secretion than the respective controls, both alone and after LPS challenge (Figure 1C). As activation of microglia represents a prominent feature in neuroinflammation associated with several NDDs, human immortalized microglial clone 3 (HMC3) cells were exposed to LPS or TNF-α to induce secretion of proinflammatory cytokines such as IL-6 and IL-8 (Dello Russo et al., 2018), however, TNF-α was used in subsequent experiments as it gave a higher response than LPS (Figures 1D,E). Neither MRx0005 nor MRx0029 reduced the production of IL-8 (Figure 1E). Of note, MRx0005 had only a marginal effect in this *in vitro* model of neuroinflammation, while MRx0029 significantly reduced IL-6 secretion in TNF-α-treated HMC3 cells. Interestingly, neither strain induced IL-6 secretion *per se* (Figure 1D) and again MRx0029 alone induced IL-8 secretion, as shown in U373 cells (Figure 1E).

### Inhibition of NF-κB Promoter Activation by MRx0005 and MRx0029

TLR4 is expressed by glial cells and binds LPS, to promote a cascade of events that culminates in activation of transcription factors including NF-κB and pro-inflammatory gene induction. To verify whether treatment with either strain would be able to interfere with NF-κB-Ap1 promoter activity induced by engagement of TLR4, HEK-TLR4 cells were treated with MRx0005 and MRx0029 alone or in combination with LPS. Both MRx0005 and MRx0029 significantly inhibited NF-κB-Ap1 promoter activation induced by LPS (Figure 1F). Interestingly, MRx0029 induced NF-κB-Ap1 promoter activation on its own, but MRx0005 did not (Figure 1F), thus suggesting that, although both strains are gram-negative bacteria, either MRx0005 interferes with the LPS signaling pathway, or a low amount/chemically different LPS is produced/shed by this strain.

### Antioxidant Capacity of MRx0005 and MRx0029

Bacteria can produce several antioxidant compounds as primary and secondary metabolites. To capture the synergistic and redox interactions among the different molecules present in the bacterial supernatants, we used three biochemical assays aimed at characterizing their antioxidant potential: the indole production assay, the total radical-trapping antioxidant parameter (TRAP) using the 2,2-diphenyl-1-picrylhydrazyl (DPPH) assay and the Trolox equivalent antioxidant capacity (TEAC) assay.

Indole derivatives are known for their antioxidant and cytoprotective activity (Estevao et al., 2010). The indole test is used to determine the ability of an organism to convert the amino acid tryptophan to form indole. The free-radical scavenging ability of antioxidants can be predicted from standard one-electron potentials by evaluating the capacity of an antioxidant to reduce an oxidant through color change. The TEAC assay measures the antioxidant capacity of a compound or a mixture of compounds.

**FIGURE 2:**
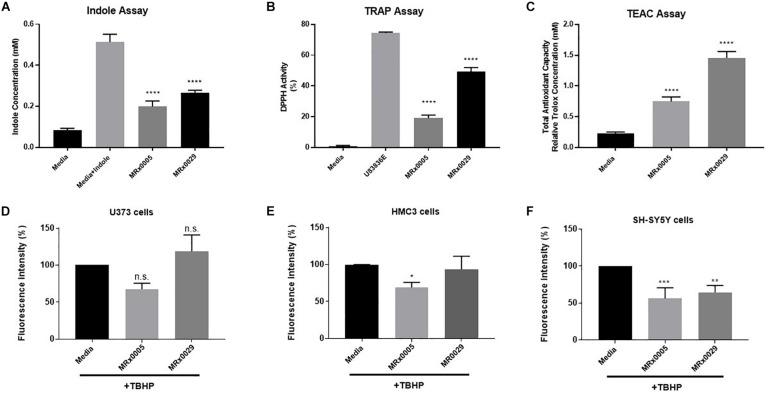
Antioxidant capacity of MRx0005 and MRx0029: Implication for neuro-protection. MRx0005 and MRx0029 bacteria were grown to stationary phase. Biochemical assays were performed as described in section “Material and Methods.” **(A)** Indole concentration (mM); **(B)** DPPH scavenging activity assay reported as percentage related to positive control compound U83836E; **(C)** Total antioxidant capacity relative to Trolox concentration (mM). Each value in the graph represents the mean of three experiments corresponding to three cultures of MRx0005 and MRx0029. Data are mean ± SEM (*n* = 3). **(D)** U373 cells, **(E)** HMC3 cells, and **(F)** differentiated SH-SY5Y cells were stained with DCFDA probe, then treated with 10% BCFS from MRx0005 and MRx0029 or media in the presence of TBHP for 2 h, followed by measurement of fluorescence intensity at Ex/Em = 485/530 nm. Data are mean ± SEM (respectively *n* = 4, *n* = 3, and *n* = 4).

Although both strains displayed clear indole-forming capacity, MRx0029 production was significantly higher than MRx0005 (0.3 and 0.2 mM, respectively, Figure 2A). The same trend was observed in relation to the ability to act as a radical scavenger in the DPPH assay and the total antioxidant capacity when compared to the lazaroid antioxidant molecule U83836E, a potent inhibitor of lipid peroxidation, or to a standard solution of Trolox, a water-soluble antioxidant derivative of Vitamin E (Figures 2B,C). Indeed, both strains showed anti-scavenging and antioxidant capacity, although MRx0029 performed better than MRx0005.

### Neuroprotection From Oxidative Stress by MRx0005 and MRx0029 Is Cell-Type Dependent

In parallel to the evaluation of the antioxidant activity of MRx0005 and MRx0029, we evaluated the ability of these strains to protect U373, HMC3 and retinoic acid (RA)-differentiated SH-SY5Y cells from reactive oxygen species (ROS) generated by treatment with Tert-Butyl Hydrogen Peroxide (TBHP). MRx0005 protected HMC3 and, albeit only partially, U373 cells from TBHP-induced ROS, while MRx0029 not only showed no protection, but appeared to slightly increase ROS production (both results were not statistically significant, Figures 2D,E). However, in differentiated SH-SY5Y cells, which recapitulate most of the features of neurons *in vitro*, both MRx0005 and MRx0029 treatment resulted in significant protection from ROS induced by TBHP (Figure 2F). These results suggested a cell preference of MRx0029 in protecting cells from hydrogen peroxide-induced ROS cytotoxicity involved in neuroinflammation and neurodegeneration.

### MRx0029 Induces a Neuron-Like Phenotype in Undifferentiated SH-SY5Y Cells

**FIGURE 3:**
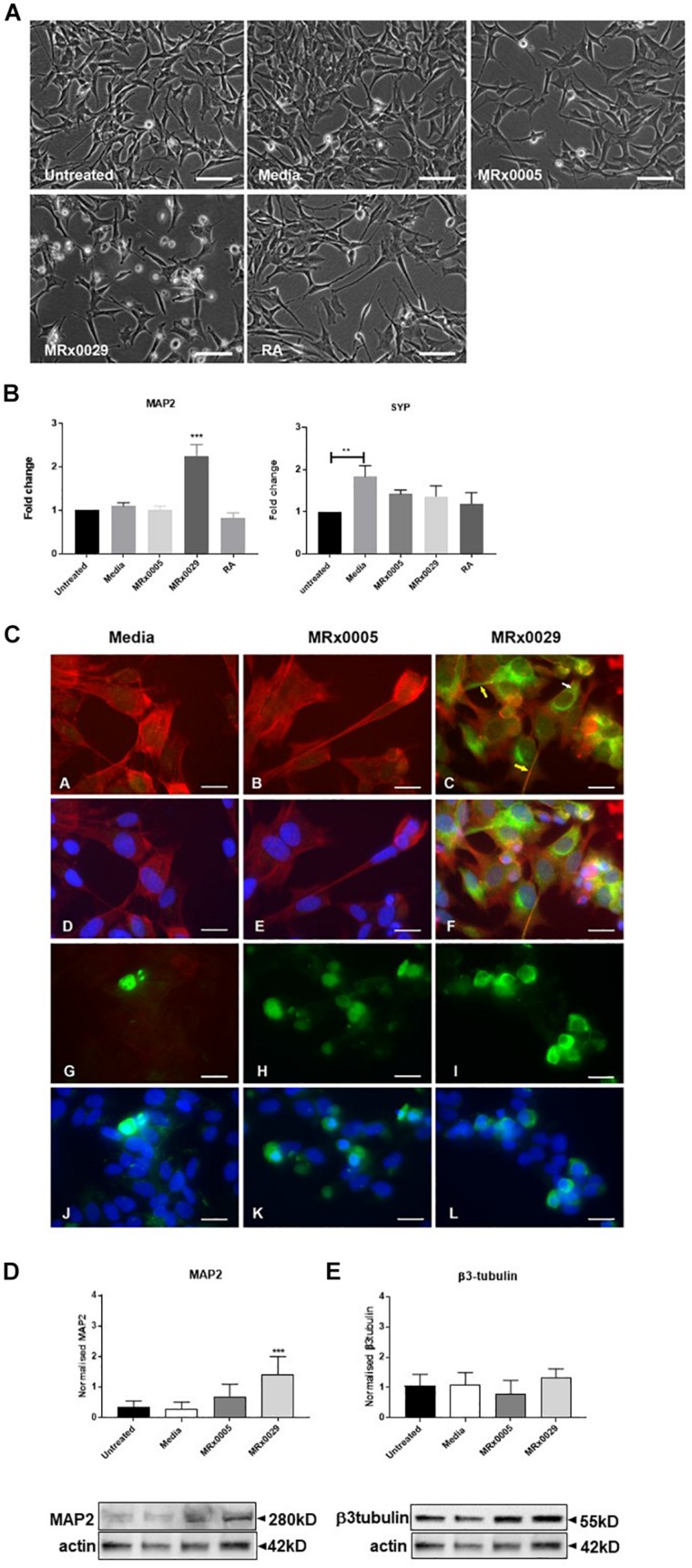
MRx0029 induced SH-SY5Y cells differentiation. Undifferentiated SH-SY5Y cells were treated with 10% MRx0005, MRx0029, media, 10 μM RA for 24 h. **(A)** Representative images using phase contrast microscope to show differences in morphology. Magnification 400×. Scale bar = 10 μm **(B)** Treated cells as described above were collected, total RNA isolated for qPCR to quantify MAP2 and SYP gene expression. Data are mean ± SEM (*n* = 3). **(C) (a–c)** Representative images of immunolabelled cells with Phalloidin (red) and MAP2 (green), **(d–f)** and merged with DAPI (blue) images; **(g–i)** β3-tubulin immunolabelled cells (green) and **(j–l)** merged with DAPI (blue) images. Magnification 630×. Scale bar = 5 μm. **(D,E)** Western blot analysis of effects of MRx0005 and MRx0029 treatment on SH-SY5Y cells. Western blot membranes were probed with antibodies to **(D)** MAP2 and **(E)** β3-tubulin. Actin was used as a loading control. Lower panels: representative blots from one of five separate experiments; upper panels: relative densitometric intensity. Data are mean ± SEM (*n* = 5).

Interestingly, treatment of undifferentiated SH-SY5Y cells with MRx0029 BCFS induced a differentiated phenotype after 24 h, as depicted in Figure 3A. MRx0029 treatment induced morphological features similar to those of cells treated with RA, a chemical commonly used for terminal differentiation of neuroblastoma cells. Qualitative cellular evaluation using bright-field phase-contrast microscopy showed that treatment with MRx0029 indeed altered the morphology of the cells. Cell bodies appeared larger and were more elongated than untreated cells, with neurite processes branching out to network with neighboring cells (Figure 3A). To further characterize the observed phenotype of MRx0029-treated cells, we investigated the expression of genes associated with neuronal differentiation, such as microtubule-associated protein 2 (MAP2) and synaptophysin (SYP). qPCR analysis revealed that MAP2 transcript was upregulated only by MRx0029. Both MRx0005 and MRx0029 slightly upregulated SYP when compared to untreated control, but not when compared to media alone. Media *per se* significantly upregulated expression of SYP in undifferentiated SH-SY5Y.

As MAP2 gene expression was upregulated solely by MRx0029 treatment (Figure 3B), we investigated whether this was reflected at protein level and how its cellular distribution in SH-SY5Y cells was affected (Figures 3C,D). To this end, we first performed immunofluorescent labeling of undifferentiated SH-SY5Y cells treated with MRx0005, MRx0029 and media and semi-quantified these results by Western blot analysis. As shown in Figure 3C, MAP2 staining was increased in MRx0029-treated cells compared to MRx0005 or media-treated cells. The staining was localized both in the cytoplasm and along the length of the extended neurites (Figure 3C panel c, yellow arrows). This pattern was also revealed with the use of another MAP2 antibody (data not shown). Western blot analysis further confirmed and semi-quantified the MAP2 upregulation induced by MRx0029 (Figure 3D). Since MAP2 is considered a marker of terminal neuronal differentiation, the results implied that MRx0029 induces neuronal differentiation in SH-SY5Y cells. Two more lines of evidence supported this notion. The staining pattern of Phalloidin in MRx0029-treated cells revealed rearrangement of the cytoskeleton with loss of flattening morphology, large cell bodies and small actin fibers and appearance of long extended neurites, enlarged growth cones and sprouting of minor neurites. Secondly, the appearance of nuclear condensation in some of the cells treated with MRx0029 (Figure 3C, panels C and F) were indicative of the pro-apoptotic phase associated with the terminal differentiation process which the cells were undergoing. We also examined the expression of the pre-terminal neuronal differentiation marker β-tubulin. Immunofluorescent labeling of SH-SY5Y cells treated with MRx0005 and MRx0029 showed an increase in staining over media-treated cells (Figure 3C, Panel G-L). However, at the protein level there was no statistical difference between the two strains (Figure 3E), even though MRx0029 appeared to have a higher expression level than media-treated cells.

### Distinct Metabolic Signatures of MRx0005 and MRx0029 Associated With Neuroprotective Phenotypes

Non-digestible carbohydrates are fermented by some members of the gut microbiota to metabolites, such as short-chain fatty acids (SCFAs), medium-chain fatty acids (MCFAs), succinate and lactate (Morrison and Preston, 2016), that can directly influence the host response to neuroinflammation, oxidative stress and cell-to-cell communication (Marcobal et al., 2013). As both MRx0005 and MRx0029 are fermentative anaerobes, we investigated whether we could identify specific metabolites from the BCFS of these strains that could be related to neuroprotection or neuroinflammation.

**FIGURE 4:**
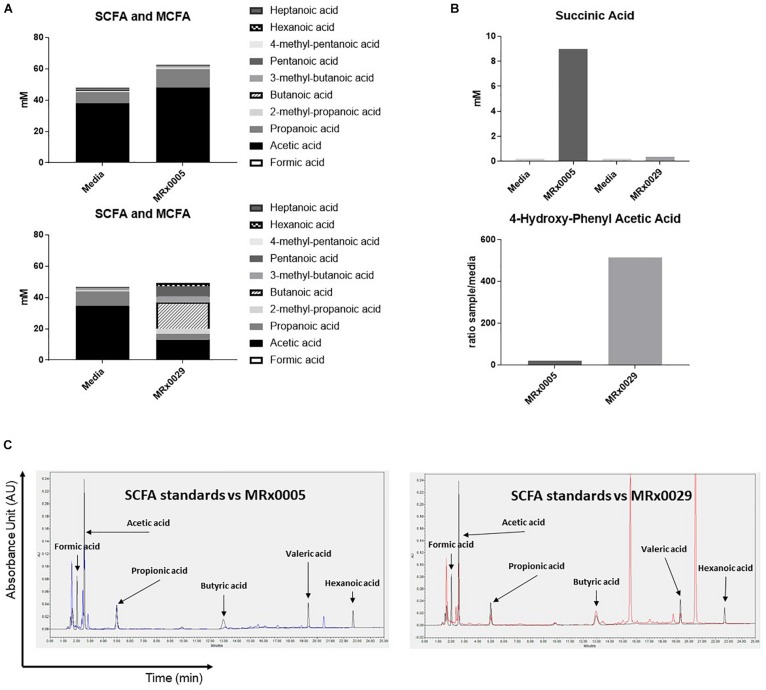
Difference in metabolites produced by MRx0005 and MRx0029. Bacteria were grown to stationary phase and processed as described in section “Materials and Methods.” YCFA^+^ was processed alongside MRx0005 and MRx0029. Authentic chemical standards were compared to retention time and mass spectra for the identified metabolites and/or based on library matching of the acquired MS spectra with the NIST library. Only metabolites that differed between the two strains are reported in this figure. **(A)** SCFA and MCFA profile (mM). **(B)** Succinic acid (mM) and 4-hydroxy-phenyl acetic acid (ratio sample/media). The data presented in **(A,B)** are from one experiment (*n* = 1). **(C)** Representative chromatograms for MRx0005 (left panel in blue) and MRx0029 (right panel in red) overlaid to a standard mix of SCFAs and MCFAs (Formic acid 10 mM, Acetic acid 20 mM, Propionic acid 10 mM, Butyric acid 10 mM, Valeric acid 10 mM, and Hexanoic acid 10 mM in black).

Fatty acid analysis, using targeted metabolomics, revealed an interesting dichotomy in the two strains: MRx0005 mainly produced acetic and propanoic acid (C2-C3), while MRx0029 produced butanoic (butyric), pentanoic (valeric) and hexanoic (caproic) acid, both in the linear and branched forms (C4-C6) (Figure 4A). Moreover, MRx0005 was found to produce high levels of succinic acid, a dicarboxylic acid with multiple metabolic activities, while the ratio of 4-hydroxy-phenylacetic acid:media was higher in MRx0029 BCFS than in MRx0005 (Figure 4B). Representative HPLC chromatograms for SCFA standards, media alone, MRx0005 and MRx0029 are reported in Figure 4C. HPLC analysis of BCFS was used to monitor the production of formic, acetic, propionic, butyric, valeric, and hexanoic acid (based on retention time and absorbance spectrum of relevant SCFAs) by MRx0005 and MRx0029. Representative chromatograms for SCFA standards overlaid to MRx0005 and MRx0029 BCFS extracted for SCFAs are reported in Figure 4C. HPLC analysis confirmed the production of acetic and propionic acid by MRx0005 and the production of butyric, valeric and hexanoic acid by MRx0029.

### Effect of SCFAs on Neuroinflammation and Neurodifferentiation

**FIGURE 5:**
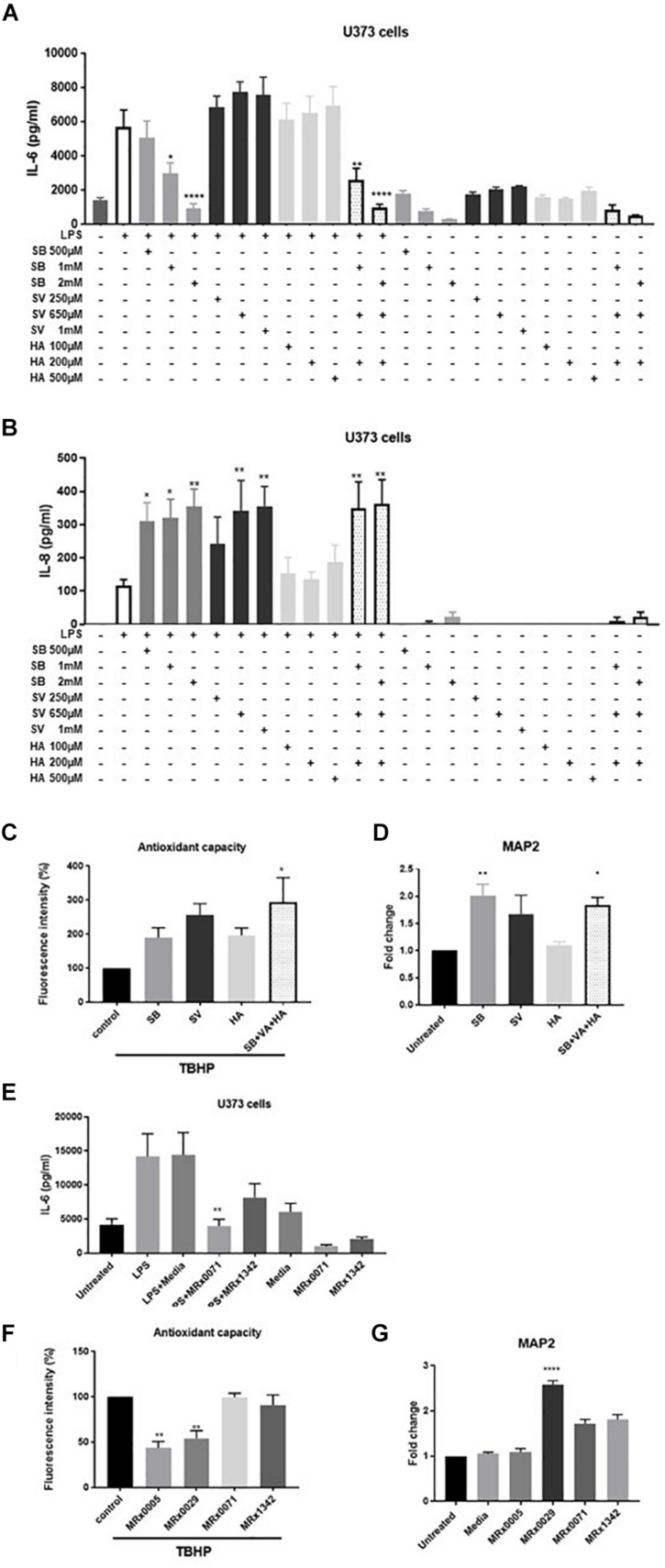
Short-chain fatty acids effect on neuroinflammation and neuroprotection. U373 cells were treated with increasing concentrations of Sodium Butyrate (SB, 500 μM–2 mM), Sodium Valerate (SV, 250 μM–1 mM), or Hexanoic acid (HA, 100–500 μM) in the presence or absence of 1 μg/ml LPS as reported in section “Materials and Methods.” Cell-free supernatants were collected 24 h after treatment and secretion of IL-6 **(A)** and IL-8 **(B)** were measured by ELISA. Differentiated SH-SY5Y were treated with 2 mM SB, 0.65 mM SV, and 0.2 mM HA or a combination of the three SCFA at the same concentration found in 10% BCFS from MRx0029. For ROS evaluation, cell treatment in the presence of 100 μM of TBHP for 2 h. Fluorescence intensity was measured at Ex/Em = 485/530 nm. **(D)** Undifferentiated cells were treated with 10% BCFS from MRx0005, MRx0029, MRx0071, and MRx1342. After 24 h, cells were collected, total RNA isolated for qPCR to quantify MAP2 gene expression. Data are mean ± SEMSH-SY5Y cells were treated with 2 mM SB, 0.65 mM SV, 0.2 mM HA or a combination of the three SCFA as described above. After 24 h, cells were collected, total RNA isolated for qPCR to quantify MAP2 gene expression. Data are mean ± SEM (*n* = 3). **(E)** U373 cells were treated with 10% BCFS from MRx0071 and MRx1342, two butyrate-producing strains from 4D pharma culture collection. Cell-free supernatants were collected 24 h after treatment and secretion of IL-6 was measured. **(F)** Differentiated SH-SY5Y cells and were stained with DCFDA and then treated with 10% BCFS from MRx0005, MRx0029, MRx0071, and MRx1342 in the presence of TBHP for 2 h, followed by measurement of fluorescence intensity at Ex/Em = 485/530 nm. **(G)** Undifferentiated SH-SY5Y (*n* = 3).

To investigate the role of SCFAs in reducing secretion of IL-6, U373 cells were treated with increasing concentrations of sodium butyrate (SB), sodium valerate (SV) and hexanoic acid (HA). The concentrations tested covered the range of concentrations measured in the BCFS for the different fatty acids and took into account the fact that only 10% of the above-mentioned supernatants was used in the cell-based assays. Interestingly, only SB inhibited LPS-induced secretion of IL-6 in U373 cells in a concentration-dependent manner (Figure 5A). Moreover, both SB and SV increased LPS-induced secretion of IL-8 in the same cells (Figure 5B), suggesting that the presence of both these SCFAs likely contributed to IL-8 induction when MRx0029 was added to the culture (Figures 5A,B). HA did not inhibit IL-6 or IL-8 secretion after challenge with LPS. None of the SFCAs tested induced *per se* secretion of IL-6 and IL-8 above the basal level (untreated cell control). In fact, only SB (at the highest concentration tested) decreased the basal level of IL-6 but not IL-8 (Figures 5A,B). The reconstituted mixture of the three SCFAs reproduced the biological activity of MRx0029 cell-free supernatant, both in the presence and absence of LPS.

To understand whether SCFAs were also involved in neuroprotection, differentiated SH-SY5Y cells were treated with TBHP in the presence or absence of SB, SV and HA. Interestingly, none of the SCFAs tested was able to protect differentiated SH-SY5Y cells from ROS (Figure 5C), suggesting that other classes of metabolites produced by our strains are responsible for their antioxidant activity on neuron-like cells. We also investigated whether SB, SV and HA were able to induce a mature phenotype in undifferentiated neuroblastoma cells. Indeed, butyrate and partially valerate exerted an activity in upregulating MAP2 gene expression in SH-SY5Y cells (Figure 5D), albeit not reaching the same fold-change shown for MRx0029 cell-free supernatant (Figures 3B, 5G). As butyrate seems to be the short-chain fatty acid with more prominent activity in both reducing IL-6 secretion from LPS-treated U373 cells and upregulation of MAP2 gene in neuroblastoma cells, two other strains, MRx0071 and MRx1342, known to be butyrate producers (Yuille et al., 2018), were tested in the same assays conducted above. As expected, MRx0071 and MRx1342 reduced LPS-induced IL-6 secretion by U373 cells (Figure 5E) without affecting the redox buffering capacity toward reactive oxygen of differentiated SH-SY5Y cells (Figure 5F). Interestingly, even though both MRx0071 and MRx1342 induced an increase in the fold change of MAP2 gene expression above the media control samples, they did not reach the significant increase seen following treatment with MRx0029, thus further supporting the hypothesis that other metabolites might contribute to the *in vitro* activity of MRx0029 (Figure 5G).

### Evaluation of the Biochemical Complexity and *in vitro* Activity of De-Proteinased MRx0005 and MRx0029 Culture Supernatants

**FIGURE 6:**
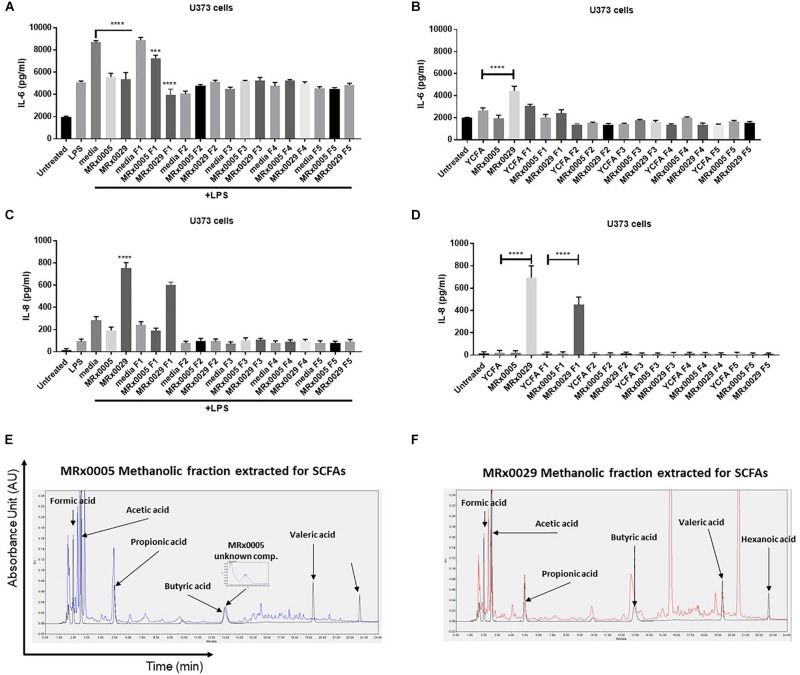
Biochemical characterization and *in vitro* evaluation of the biological activity of MRx0005 and MRx0029 culture supernatant fractions. BCFS from three biological replicates from MRx0005 and MRx0029 were mixed with different solvents of increasing polarity and extracts used in cell-based assays. 10% BCFS, media alone, five different fractions and blank media treated with the same solvents used to prepare the fractions were added to U373 cells in the presence or absence of LPS as described in section “Materials and Methods.” Cell-free supernatants were collected 24 h after treatment and secretion of IL-6 and IL-8 was measured by ELISA. IL-6 secretion measured in U373 cells **(A)** pre-treated with 1 μg/ml LPS or in the absence of LPS **(B)**; IL-8 secretion measured in U373 cells **(C)** pre-treated with 1 μg/ml LPS or in the absence of LPS **(D). (E,F)** The MeOH fractions from MRx0005 and MRx0029 were analyzed by HPLC for SCFAs band MCFAs. Representative examples of HPLC chromatograms are shown for MRx0005 and MRx0029. The methanolic fractions for MRx0005 **(E)** and MRx0029 **(F)** were extracted for SCFAs and MCFAs and then overlaid to a standard mix of SCFAs and MCFAs (Formic acid 10 mM, Acetic acid 20 mM, Propionic acid 10 mM, Butyric acid 10 mM, Valeric acid 10 mM, and Hexanoic acid 10 mM in black). The chromatograms highlight the lack of butyric, valeric and hexanoic acid in the MRx0005 methanolic fraction and their presence in the MRx0029 methanolic fraction.

BCFS are complex mixtures of secondary metabolites, including peptides and proteins. With the aim of further confirming whether the anti-neuroinflammatory activity of MRx0029 and MRx0005 activity was mainly due to SCFAs, BCFS were sequentially extracted with different solvents of increasing polarity. HPLC analysis of the de-proteinased crude extracts (hexane, F5; diethyl ether, F4; ethyl acetate, F3; acetonitrile, F4; methanol, F1) of MRx0005 and MRx0029 supernatants was conducted to analyse the biochemical complexity of the stationary phase BCFS of the two strains as well as to sub-fractionate compounds based on polarity and solubility. HPLC analysis confirmed the selective extraction and crude fractionation of compounds present in the de-proteinased supernatants (data not shown). U373 cells were treated with the unfractionated BCFS or the different fractions from MRx0005 and MRx0029 both in the presence and absence of LPS. BCFS were collected 24 h after treatment and analyzed by ELISA for IL-6 and IL-8 secretion. The methanolic fraction F1 of MRx0029 decreased IL-6 production and appeared to recapitulate the activity of the unfractionated supernatant, further indicating that the presence of butyrate is associated with the anti-inflammatory activity of this strain. The unfractionated MRx0005 supernatant had a superior effect on IL-6 inhibition compared to the methanolic fraction F1 of this strain (Figure 6A). The partial loss of activity might be ascribable both to the removal of a proteinaceous bioactive component of the supernatant or be related to the decrease in availability of a compound like succinic acid due to the chemical processes in place to separate the different fractions. In the absence of LPS, only the unfractionated MRx0029 retained its ability to induce IL-6 (Figure 6B). As expected, unfractionated MRx0029 induced IL-8 secretion in U373 cells both in the presence and absence of LPS, and the same activity was produced by the methanolic fraction F1, thus reiterating the important role of butyric and valeric acid in IL-8 production by these neuronal cells (Figures 6C,D). Moreover, HPLC analysis confirmed the selective extraction and fractionation of compounds present in the de-proteinased extracts, as well as corroborated the presence of acetate and propionate in the methanolic fraction of MRx0005 and butyrate, valerate and hexanoate in the methanolic fraction of MRx0029 (Figures 6E,F).

## Discussion

Neuroinflammation is a common factor in neurodegenerative diseases such as Parkinson’s disease (PD) and Alzheimer’s disease (AD) (Stephenson et al., 2018). Both genetic (e.g., in rare Mendelian forms of PD and AD) and environmental triggers (e.g., pollutants, neurotoxic metals) play a pivotal role in inducing sustained inflammation in different regions of the brain and in the periphery. Changes in the composition of the human gut microbiome may contribute to neuroinflammation and have indicated the importance of the so-called gut-brain axis in disease setting and progression (Rea et al., 2016). Alterations in gut barrier permeability are associated with host immune responses to molecules of gut bacterial origin, such as lipopolysaccharides (LPS), which are able to reach the systemic circulation and induce a proinflammatory response (Hayes et al., 2018). Cytokines and chemokines are involved in the repair of damaged tissues as well as in the amplification of the proinflammatory response (Charo and Ransohoff, 2006). Proinflammatory cytokines such as IL-6 and IL-8 have been detected in the serum of PD and AD patients and correlate with disease progression and decline of cognitive functions (Reale et al., 2009).

We first investigated the ability of individual members of the gut microbiome to influence neuroinflammatory processes. For this purpose, human glioblastoma astrocytoma U373 cells were treated with LPS, and IL-6 and IL-8 secretion was measured in the cell supernatants. As we hypothesized that bacterial metabolites might confer protection in neuroinflammatory processes, we treated the U373 cells with bacterial supernatants from a panel of bacterial strains. Out of 50 bacterial supernatants tested, MRx0005 and MRx0029 showed a remarkable activity in reducing IL-6 secretion by U373 cells after treatment with LPS.

MRx0005 (*P. distasonis*) and MRx0029 (*M. massiliensis*) are strains from the 4D Pharma culture collection originally isolated from the fecal samples of healthy human donors which we have identified as having protective potential. *P. distasonis* is an obligately anaerobic Gram-negative, rod-shaped, non-spore forming and non-motile bacterial species from the Bacteroidetes phylum (Sakamoto and Benno, 2006). *P. distasonis* is a common member of the commensal microbiota in the human intestine and strains of this species have been studied for their beneficial effects on Multiple Sclerosis (Cekanaviciute et al., 2017). *M. massiliensis* is an obligately anaerobic Gram-negative coccobacillus, which is non-spore forming, non-motile and is a member of the Firmicutes phylum (Padmanabhan et al., 2013). Together with the phylum Bacteroidetes, Firmicutes are a dominant part of the human gut microbiota (Turnbaugh and Gordon, 2009).

The strong reduction in IL-6 secretion by U373 cells treated with MRx0005 and MRx0029 cell-free supernatants together with the intrinsic biotherapeutic potential of these two species prompted us to further characterize these two strains. U373 cells produced IL-8 in response to LPS. Like IL-6, IL-8 is a pro-inflammatory cytokine whose presence in the serum is associated with cognitive dysfunction in the elderly (Baune et al., 2008). Interestingly, IL-8 can also work as a neurotrophic factor by promoting survival of hippocampal neuronal cultures (Araujo and Cotman, 1993). We showed that MRx0005, but not MRx0029, was able to reduce IL-8 production. This could be of relevance in relation to the ability of MRx0029 to induce *per se* significant levels of IL-8.

As LPS is a ligand for TLR4, a similar experimental setting was used with the HEK-TLR4 reporter cell line. While both strains reduced NF-kB activation after treatment with LPS, only MRx0029 induced NF-kB activation *per se*, thus further confirming the observations made in U373 cells. Further work is needed to understand whether the potency of the response might be related to different isoforms of LPS or the quantity secreted by the two strains.

Oxidative stress is associated with cellular neurodegenerative processes in different regions of the brain (Chen et al., 2012). As bacteria can produce different antioxidant metabolites and detoxifying enzymes (Li et al., 2003; Lin et al., 2014), we evaluated the intrinsic scavenging and total antioxidant capacity of both strains by measuring the production of indole, the total radical-trapping antioxidant parameter (TRAP) and the antioxidant capacity of cell-free supernatants in relation to Trolox. Overall, MRx0029 showed a superior antioxidant capacity than MRx0005. However, only MRx0005 conferred protection from reactive oxygen species (ROS) in glioblastoma U373 cells and microglia HMC3 cells. Interestingly, differentiated neuroblastoma SH-SY5Y cells treated with cell-free supernatants showed that MRx0029 was as good as MRx0005 in protecting these cells from TBHP toxicity, suggesting a surprisingly specific tropism of MRx0029 for neuron-like cells.

Differentiated SH-SY5Y cells recapitulate many of the biochemical and functional properties of neurons (Agholme et al., 2010; Forster et al., 2016) and are a commonly used model for NDDs. When undifferentiated SH-SY5Y cells were treated with cell-free supernatants from MRx0005 and MRx0029, microscopic examination revealed that SH-SY5Y cells treated with MRx0029 resembled a neuron-like phenotype, with multiple elongated processes on an oblong body. Gene expression of markers distinctive for differentiated neurons, such as microtubule-associated protein 2 (MAP2) and synaptophysin (SYP), indeed confirmed that MRx0029 induced a differentiated phenotype in neuroblastoma cells. MAP2 upregulation was observed both at gene and protein levels. Further, immunofluorescent labeling revealed that MAP2 localized in the neurites and dendrites as well as in perikarya of mature neurons (Tanapat, 2013). Phalloidin staining indicated a mature neuron-like rearrangement of the cytoskeleton, so we investigated the expression and staining pattern of β3-tubulin, another neurogenesis-associated differentiation marker which plays a role in neuron-specific intracellular structural and transport processes (Pellegrini et al., 2017). Once again, MRx0029 but not MRx0005 induced an upregulation at protein level of β3-tubulin, albeit not to a significant level.

The tandem increase of protein expression of β3-tubulin and more importantly of MAP2 by MRx0029 further strengthens our hypothesis of a tropism of MRx0029 toward a mature phenotype, suggesting that MRx0029 metabolites might alter microtubule dynamics in neuron-like cells. Moreover, as MAP2 promotes neurite outgrowth, which play a major role in re-networking of damaged neurons and synaptogenesis, MAP2 expression might go beyond being a marker of neuronal differentiation and indicate “neuronal re-wiring” associated with the therapeutic outcome of neuropathological disease (Abdanipour et al., 2015).

Overall, we have demonstrated that MRx0005 and MRx0029 affect host function differently in different types of brain cells, with distinct effects on inflammation and neuronal cells. The caveat here is that the cells we have used in this study are either immortalized or derived from tumors. This choice was made to assure a reproducibility of the data at this stage of the project. More investigation using primary microglia cells and neurons are undoubtedly necessary and planned to further understand the biological meaning behind our findings.

We postulate that the effects observed on neuroinflammation and oxidative stress are mediated by bacterial production of different classes of metabolites. SCFAs produced by gut bacteria are typically recognized as positive modulators of host functions. SCFAs mediate water and sodium intake by epithelial cells and play an important role in the proliferation, differentiation and regulation of epithelial cells (Wang et al., 2019). For example, propionate and butyrate participate in the preservation of mucosal integrity, thwart the proliferation of pathogens in the gut and have been shown to modulate energy metabolism through the gut-brain axis (den Besten et al., 2013; van der Beek et al., 2017; Li et al., 2018). Valeric acid interferes with β-amyloid peptides 1-40 and 1-42 fibril formation *in vitro* and prevents α-synuclein monomers from pairing up and aggregating into fibrils (Ho et al., 2018). Hexanoic acid has been shown to reduce the colonization and dysbiotic expansion of potentially pathogenic bacteria in the gut (Van Immerseel et al., 2004). Succinic acid plays a neuroprotective role in oxidative phosphorylation, a key step for synaptic trafficking of proteins to proximal and distal regions (Budd and Nicholls, 1998). It also augments mitochondrial activity and together with BDNF supports vulnerable neurons in neurodegenerative disorders (Ferro et al., 2017).

MRx0029 is a strong producer of butyric, valeric and hexanoic acid and we investigated the ability of these SCFAs to decrease neuroinflammation in glioblastoma cells and oxidative stress in neuron-like cells. They were tested as single SCFAs and in combination at the same concentrations present in the cell-free supernatant. Interestingly, only butyric acid was able to reduce IL-6 secretion in the presence of LPS in a concentration-dependent manner. Neither valeric nor hexanoic acid inhibited IL-6 or IL-8 secretion in the presence of LPS. The combination of the three SCFAs reconstituted at the same concentration measured in MRx0029 supernatant recapitulated the activity of the supernatant, suggesting that butyrate drove the anti-inflammatory activity of MRx0029. However, butyrate and valeric acid were only partially involved in the ability of MRx0029 to induce a mature phenotype in neuroblastoma cells. It has been reported that medium-chain fatty acids can promote neurite outgrowth (Kamata et al., 2007).

To simplify the complexity of the supernatant and assess the activity of the SCFAs, the cell-free supernatants from MRx0005 and MRx0029 were extracted with solvents of increasing polarity. Only the methanolic fraction of MRx0029 containing butyric, valeric and hexanoic acid produced the same effects on U373 cells as described above. Of note, the methanolic fraction of MRx0005 containing acetate and propionate only partially recapitulated the anti-inflammatory activity of MRx0005, which might be due to the removal of proteinaceous bioactive molecules or to the chemical treatment resulting in the potential generation of esters of fatty acids such as succinate. Our theory is that is the synergistic activity of SCFAs and other metabolites produced by MRx0029 creates the neurotrophic activity of this strain and supports its potential therapeutic use as an LPB in the treatment of NDDs.

MRx0029 is a histone deacetylase (HDAC) inhibitor through its production of butyrate, as shown in a research paper recently published by our group (Yuille et al., 2018). Wakade and Chong investigated the neuroprotective mechanisms of butyric acid in the context of Parkinson’s disease by analyzing the relationship between the niacin receptor and dopamine levels (Wakade and Chong, 2014). Butyric acid has the potential to beneficially impact Parkinson’s symptoms by reducing inflammation, increasing dopamine synthesis (by improving the amount of free niacin that is available for dopamine synthesis) and boosting mitochondrial function to provide cells with more energy (Donohoe et al., 2011; Bourassa et al., 2016). Our findings highlight how the activity of a specific combination of SCFAs can affect neuropathological aspects of NDDs and how the use of LBPs rather than the single purified metabolite could be therapeutically successful.

In summary, this study provides compelling evidence of how using our *in vitro* screening platform we have identified two gut-derived bacteria that can modulate relevant cell types targeted by neuroinflammation and oxidative stress in NDDs. The strains *P. distasonis* MRx0005 and *M. massiliensis* MRx0029 both have an anti-inflammatory signature, however, MRx0029 seems to preferentially protect neurons from cytotoxicity induced by oxidative stress. More investigations using relevant *in vitro* and *in vivo* models are underway to fully dissect the molecular pathways behind the therapeutic use of these bacteria strains in neurodegenerative diseases.

## Data Availability

16S gene sequences for MRx0005 and MRx0029 are disclosed in International Patent Publication Nos. WO2018/229189 and WO2018/229216, respectively, filed by 4D Pharma Research Ltd. The data supporting the findings in this paper are available within the article and its Supplementary Information Files.

## Ethics Statement

Ethical approval for collection of faecal samples from healthy human donors was granted from the West of Scotland Research Ethics Committee (Ref. 15/WS/0277). The biological samples have been obtained with any necessary informed written consent from the volunteering participants.

## Author Contributions

*In vitro* experiments: AE, SA, and AB designed the experiments; SA, PF, NV, MG, SB, GB-A, and MD performed the majority of the *in vitro* experiments; SR, HD, and AB validated, performed, and analyzed the microbiological-related data output; AE coordinated and managed the research project; AE, SA, PF, and AB analyzed the data. IM oversaw the overall research plan. AE wrote the manuscript with the assistance of SA and AB. All authors have read and commented on the manuscript and have approved the final version of the manuscript.

## Conflict of Interest Statement

All authors were employees of (or in the case of MID, seconded full-time to 4D Pharma Ltd.) 4D Pharma Research Ltd., while engaged in the research project. AE, SA, PF, and IM were named as inventors in all or some the patents listed above.
