# Supplementary material for: In vitro Characterization of Gut Microbiota-Derived Bacterial Strains With Neuroprotective Properties
Source: Front Cell Neurosci. 2019 Sep 20;13:402. doi: 10.3389/fncel.2019.00402 (PMC6763572; doi:10.3389/fncel.2019.00402)
Supplement: Supplementary file 1 [file Table_1.pdf]

Supplementary Table S1

List of primers used for *in vitro* gene expression analysis by qPCR.

Gene ID	Forward sequence	Reverse sequence
**ACTB**	GATCAAGATCATTGCTCCTC	TTGTCAAGAAAGGGTGTAAC
**GAPDH**	GGTATCGTGGAAGGACTCATG	ATGCCAGTGAGCTTCCCGTTC
**MAP2**	CTCAGCACCGCTAACAGAGG	CATTGGCGCTTCTCTCCTC
**SYP**	CTCGGCTTTGTGAAGGTGCT	GGCTTCATGGCATCAACTTCA
